# Estrogen administered after cardiac arrest and cardiopulmonary resuscitation ameliorates acute kidney injury in a sex- and age-specific manner

**DOI:** 10.1186/s13054-015-1049-8

**Published:** 2015-09-18

**Authors:** Mizuko Ikeda, Thomas Swide, Alexandra Vayl, Tim Lahm, Sharon Anderson, Michael P. Hutchens

**Affiliations:** Department of Anesthesiology and Perioperative Medicine, Oregon Health and Science University, 3181 SW Sam Jackson Park Road, Portland, OR 97239 USA; Division of Pulmonary, Critical Care, Sleep and Occupational Medicine, Indiana University School of Medicine, Joseph E. Walther Hall, R3 C400 980 W. Walnut St., Indianapolis, IN 46202 USA; Richard L. Roudebush VA Medical Center, 1481 W 10th St, Indianapolis, IN 46202 USA; Department of Internal Medicine, Division of Nephrology and Hypertension, Oregon Health & Science University, 3181 SW Sam Jackson Park Road, Portland, OR 97239 USA

## Abstract

**Introduction:**

There is a sex difference in the risk of ischemic acute kidney injury (AKI), and estrogen mediates the protective effect of female sex. We previously demonstrated that preprocedural chronic restoration of physiologic estrogen to ovariectomized female mice ameliorated AKI after cardiac arrest and cardiopulmonary resuscitation (CA/CPR). In the present study, we hypothesized that male mice and aged female mice would benefit from estrogen administration after CA/CPR. We tested the effect of estrogen in a clinically relevant manner by administrating it after CA/CPR.

**Methods:**

CA/CPR was performed in young (10–15 weeks), middle-aged (43–48 weeks), and aged (78–87 weeks) C57BL/6 male and female mice. Mice received intravenous 17β-estradiol or vehicle 15 min after resuscitation. Serum chemistries and unbiased stereological assessment of renal injury were completed 24 h after CA. Regional renal cortical blood flow was measured by a laser Doppler, and renal levels of estrogen receptor alpha (ERα) and G protein-coupled estrogen receptor (GPER) were evaluated with immunoblotting.

**Results:**

Post-arrest estrogen administration reduced injury in young males without significant changes in renal blood flow (percentage reduction compared with vehicle: serum urea nitrogen, 30 %; serum creatinine (sCr), 41 %; volume of necrotic tubules (VNT), 31 %; *P* < 0.05). In contrast, estrogen did not affect any outcomes in young females. In aged mice, estrogen significantly reduced sCr (80 %) and VNT (73 %) in males and VNT (51 %) in females. Serum estrogen levels in aged female mice after CA/CPR were the same as levels in male mice. With age, renal ERα was upregulated in females.

**Conclusions:**

Estrogen administration after resuscitation from CA ameliorates renal injury in young males and aged mice in both sexes. Because injury was small, young females were not affected. The protective effect of exogenous estrogen may be detectable with loss of endogenous estrogen in aged females and could be mediated by differences in renal ERs. Post-arrest estrogen administration is renoprotective in a sex- and age-dependent manner.

## Introduction

Acute kidney injury (AKI) is a common complication of perioperative illness, and mortality is up to 70 %. In noncardiac surgical patients, the incidence is 1 %, conferring a seven- to eight-fold increment in mortality. The most common cause of hospital-acquired AKI is whole-body hypoperfusion [1–5], which is also independently associated with perioperative AKI.

Overall, men are more likely to experience AKI than women [6, 7]. Clinical data suggest that low estrogen states are associated with increased risk of AKI [8, 9]. Data from animal models indicate that sex hormones mediate AKI; androgen and testosterone enhance the susceptibility to AKI, and estrogen is a protective factor [10, 11]. We previously demonstrated in mice that females enjoy relative protection from AKI after whole-body ischemia-reperfusion injury, removal of physiologic estrogen worsens AKI, and preprocedural restoration of physiologic estrogen to ovariectomized female mice restores the protected state [12, 13].

Although estrogens have been studied widely, whether estrogen is efficacious when administered after whole-body ischemia-reperfusion and whether such protection can be observed in males as well as females with intact ovaries have not been determined. Therefore, to gain insights into the role of estrogen as a treatment option, we determined whether a single injection of estrogen, administered on recovery from cardiac arrest and cardiopulmonary resuscitation (CA/CPR), improves AKI in both sexes and whether the effect is dependent on renal blood flow. It is relevant to administer estrogen in acute phase after ischemic insult in the aspect of investigating estrogen as a therapeutic agent since clinically CA is frequently unheralded, and therefore therapy is applied following resuscitation.

Clinical trials of ischemia-protective strategies proven in mice may have failed in the past because of the reliance on young mice [14-16]. Both experimental and clinical studies demonstrate the strong linkage between aging and AKI [17-19]. Clinical data suggest that women enjoy protection from AKI in general but not when undergoing cardiac and vascular surgery, settings in which patients are older than the overall surgical population [20]. This bimodal risk distribution may be a phenomenon of the menopause and changing exposure to estrogen with age. However, few studies have addressed effect modifications of aging on the association between sex and AKI. Therefore, other important questions are whether the advantage of females against AKI after whole-body ischemia is lost with age and whether this is modulated by estrogen.

Together, the major aims of the present study were (1) to determine whether post-arrest administration of estrogen reduces AKI following CA/CPR in both sexes and (2) to investigate whether aged females, the clinical population with greatest AKI risk, lose the beneficial effect of female sex or benefit from post-arrest administration of estrogen or both. A further aim was to determine whether estrogen receptor (ER) populations in the kidney, unaffected by pre-arrest hormonal manipulation, might change with age.

## Methods

All experimental protocols involving animals were conducted at Oregon Health & Science University in Portland, Oregon. All animal experiments were conducted in conformity with the National Institutes of Health guidelines for the care and use of animals in research and were specifically approved by the Oregon Health & Science University Institutional Animal Care and Use Committee.

### Animals and experimental groups

Male and female C57BL/6 mice were obtained from Charles River Laboratories (Boston, MA, USA). We used young mice (10–15 weeks), middle-aged mice (43–48 weeks), and aged mice (78–87 weeks). Experimental animals were randomized to 17β-estradiol or vehicle treatment and subsequently underwent experimental procedures.

### *In vivo* whole-body ischemia-reperfusion (CA/CPR)

We performed normothermic CA/CPR as previously described [12, 13, 21, 22] Briefly, mice were anesthetized with 4 % isoflurane in a 2:1 air-to-oxygen mixture, and anesthesia was maintained with 1 %–2 % isoflurane. After tracheal intubation with a 22-guage Teflon catheter (BD, Franklin, NJ, USA), animals were mechanically ventilated and the electrocardiogram (EKG) was monitored continuously. For drug administration, a PE-10 catheter was inserted into the right internal jugular vein. Rectal temperature was maintained 37 ± 5 °C throughout the experiment with a digitally controlled lamp (Cole Parmer, Vernon Hills, IL, USA). CA was induced with 50 μl of 0.5 M potassium chloride and confirmed by isoelectric EKG signal and absence of visible cardiac contraction on the chest wall. At onset of CA, the endotracheal tube was disconnected. Seven and a half minutes after onset of CA, mechanical ventilation resumed. Eight minutes after onset of CA, chest compressions were delivered by a finger at a rate of 300 per minute, and epinephrine in 0.9 % sodium chloride solution, 11–16 μg (0.7–1.0 ml), was administered intravenously. Return of spontaneous circulation (ROSC) was confirmed by reappearance of electrical activity on the EKG and visible cardiac contractions on the chest wall. Animals were extubated when their spontaneous respiratory rate reached more than 60 breaths per minute and then were moved to a recovery cage.

### Preparation of 17β-estradiol and correspondent vehicle

A previous study performed in the same laboratory demonstrated that injection of 0.5 μg of 17β-estradiol resulted in a peak of serum estradiol levels shortly after administration followed by a decrease to physiologic levels within 1 h in male C57BL/6 mice [23]. We therefore administered 0.5, 5, and 10 μg of 17β-estradiol to investigate whether any renoprotection is dose-dependent. Water-soluble cyclodextrin-encapsulated 17β-estradiol (E4389; Sigma-Aldrich, St. Louis, MO, USA) was dissolved in 0.9 % sodium chloride solution to be the desired dose in 100 μl. Each vehicle control was rendered so as to contain the same amount of hydroxypropyl cyclodextrin as its corresponding 17β-estradiol treatment.

### Regional renal cortical blood flow measurement

We measured regional renal cortical blood flow (RRCBF) in the peri-arrest period. A laser Doppler flow probe (Moor Instruments, Wilmington, DE, USA) was placed perpendicular to the surface of the right kidney via a flank incision and optimally positioned for maximal signal. Laser Doppler flow was measured and recorded 5 min before CA, immediately before CA, every minute during CA, and every 5 min after CA for 45 min after ROSC for each animal treated with 17β-estradiol or vehicle at 15 min after ROSC. Fifty minutes after ROSC, arterial blood was aspirated from the left ventricle for blood gas assessment.

### Transcardial perfusion and tissue sampling

Twenty-four hours after CA/CPR, thoracotomy was performed under deep anesthesia. After blood sampling, mice were perfused with saline, and the right kidney was snap-frozen in liquid nitrogen and stored at −80 °C until immunoblotting. Subsequently, mice were perfused with 4 % paraformaldehyde and then the left kidney was harvested for histological assessment.

### Evaluation of serum urea nitrogen, serum creatinine, and serum estradiol levels

A point-of-care enzyme-coupled analyzer (Abaxis Medical Diagnostics, Union City, CA, USA) was used to measure serum urea nitrogen (sUN) and serum creatinine (sCr). 17β-estradiol concentrations were measured by using radioimmunoassay as previously described [12]. The GDN 244 antibody was used for radioimmunoassay [24]. The overall inter-assay variation for 17β-estradiol extraction radioimmunoassay was less than 15 %, and the intra-assay variation was less than 12 %.

### Histological evaluation of tubular injury

An observer blinded to treatment and sex assessed histological damage by using rigorous unbiased stereology. After fixation, 6-μm sagittal sections from the kidney were sampled 160 μm apart and then stained with Flouro-Jade B (Histo-Chem, Jefferson, AK, USA), which stains necrotic cells bright green. Histological damage was determined in accordance with the Cavalieri principal of unbiased stereology by using Visiopharm Integrator Software (Visiopharm, Hørsholm, Denmark) and delineating the total kidney area and applying superimposed grids consisting of two marks in a fixed relation (4:64) randomly onto a field of view. The estimated volume fraction of necrotic tubules was calculated as follows:

$$ \mathrm{Volume}\ \mathrm{necrotic}\ \mathrm{tubules}\ \left(\mathrm{V}\mathrm{N}\mathrm{T}\right)={\sum}_{\mathrm{Marks}\ \left(\mathrm{necrotic}\ \mathrm{tubules}\right)}/\ {\sum}_{\mathrm{Marks}\ \left(\mathrm{kidney}\right)}\times 16 $$

The sampling intensity was decided according to our previous study to obtain precise estimation with coefficient of error of less than 0.1 by using the formula of Gundersen et al. [25]. The coefficients of error for the necrotic tubules and the reference area in our study were 0.08 and 0.05, respectively.

### Immunoblotting

Expression of ERα and G protein-coupled estrogen receptor (GPER) in homogenates of the entire right kidney obtained 24 h after CA/CPR were measured by using rabbit polyclonal anti-ERα (clone HC-20; Santa Cruz Biotechnology, Inc., Dallas, TX, USA) and anti-GPER (clone N-15; Santa Cruz Biotechnology, Inc.) antibodies. To eliminate any effect of exogenously administered estradiol, this assay was performed only in vehicle-treated mice. Mouse monoclonal anti-vinculin (clone cp74; CalBiochem/EMD Millipore, Billerica, MA, USA) was used as loading control. Secondary antibodies were anti-mouse horseradish peroxidase (HRP) and anti-rabbit-HRP from GE Healthcare (Little Chalfont, Buckinghamshire, UK) and Cell Signaling Technology (Danvers, MA, USA), respectively. Tissue was homogenized in ice-cold radioimmunoprecipitation assay buffer (ThermoFisher Scientific, Waltham, MA, USA) containing protease inhibitor cocktail (Sigma-Aldrich) and PhosSTOP phosphatase inhibitor cocktail (Roche, Indianapolis, IN, USA). Protein concentration was measured by using the BCA protein assay (ThermoFisher Scientific). Western blots were performed as previously described [26]. Primary antibodies were used at a dilution of 1:50 (ERα), 1:500 (GPER), or 1:2000 (vinculin) in 5 % bovine serum albumin (BSA) in tris-buffered saline with Tween-20 (TBST) (25 mM Tris, 1 M NaCl, 1 % Tween 20). Secondary antibodies were diluted 1:2000 (ERα), 1:5000 (GPER), or 1:15,000 (vinculin) in 5 % BSA in TBST.

### Statistical analysis

Statistical significance was inferred from a *P* value of less than 0.05. Two-group two-treatment analyses were performed by using two-way analysis of variance with *post hoc* Sidak’s test. Categorical outcomes were assessed by using Fisher’s exact test or chi-squared test, as appropriate. Analysis of differences in estrogen expression between young and aged mice was performed via unpaired *t* test. All results are presented as mean ± standard deviation. Analysis was performed with Prism 6.0 software (GraphPad Software, Inc., La Jolla, CA, USA).

## Results

### Male mice are more vulnerable to renal injury than female mice after CA/CPR

**Fig. 1:**
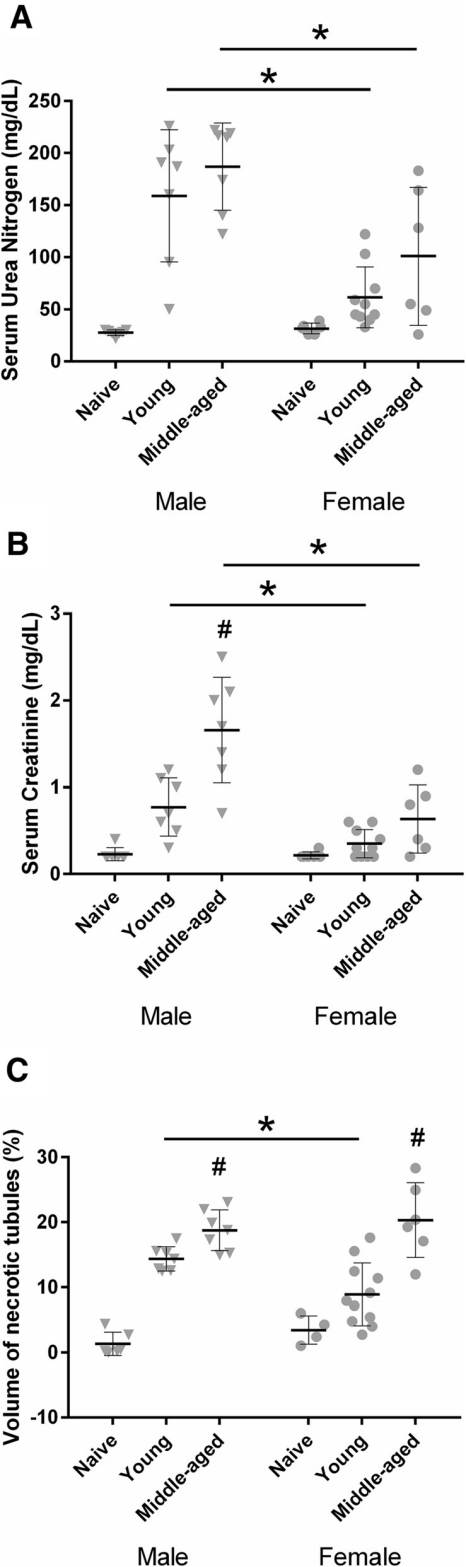
Cardiac arrest and cardiopulmonary resuscitation (CA/CPR) caused renal injury in a sex- and age-specific manner. Blood and tissue samples were collected 24 h after CA/CPR in young (10–15 weeks) or middle-aged (43–48 weeks) male or female mice. CA/CPR caused renal injury in a sex- and age- specific manner. In young animals, female sex reduced serum urea nitrogen (**a**), serum creatinine (**b**), and necrotic tubules (**c**). In middle-aged animals, female sex reduced serum urea nitrogen (**a**) and serum creatinine (**b**) but not necrotic tubules (**c**). Data are presented as mean ± standard deviation, n = 6–10 per group. *P < 0.05 versus same age different sex, ^#^ *P* < 0.05 versus same sex different age. Data were analyzed by using two-way analysis of variance with *post hoc* Sidak’s test. Aging did not alter baseline renal function in naïve mice; therefore, we combined young and middle age naïve mice

To evaluate the severity of AKI after CA/CPR according to sex and age, male and female C57BL/6 young and middle-aged mice were subjected to 8 min of CA. Twenty-four hours after CPR, a blood sample was collected and kidney was harvested. Figure 1 summarizes the renal injury 24 h after CA/CPR according to sex and age. CA/CPR caused renal injury in a sex-specific manner. In surgically naïve animals, sUN and sCr were equivalent between male and female (sUN: 28 ± 2.8 versus 30 ± 3.5, *P* = 0.17; sCr: 0.23 ± 0.08 versus 0.22 ± 0.04, *P* = 0.73; and volume of necrotic tubules (VNT): 1.3 ± 1.8 versus 3.4 ± 2.2, *P* = 0.15, male versus female). Young males developed greater renal injury than young females after CA/CPR (sUN: 159 ± 64 versus 62 ± 29, *P* < 0.01; sCr: 0.77 ± 0.33 versus 0.35 ± 0.17, *P* < 0.01; and VNT: 14 ± 1.9 versus 8.9 ± 4.8, *P* < 0.01, male versus female). In middle-aged males, functional renal injury was greater than that of females after CA/CPR (sUN: 187 ± 42 versus 101 ± 66, *P* < 0.01 and sCr: 1.7 ± 0.6 versus 0.63 ± 0.4, *P* < 0.01). Tubular necrosis, however, was equivalent between male and female middle-aged mice (VNT: 19 ± 3.1 versus 20 ± 5.8, *P* = 0.76). In summary, young males suffer greater renal injury than young females after CA/CPR. Middle-aged males also demonstrate more severe post-ischemic renal dysfunction than middle-aged females; however, the severity of tubular necrosis is the same.

### Dose-dependent renoprotection by post-arrest administration of 17β-estradiol in young males

**Fig. 2:**
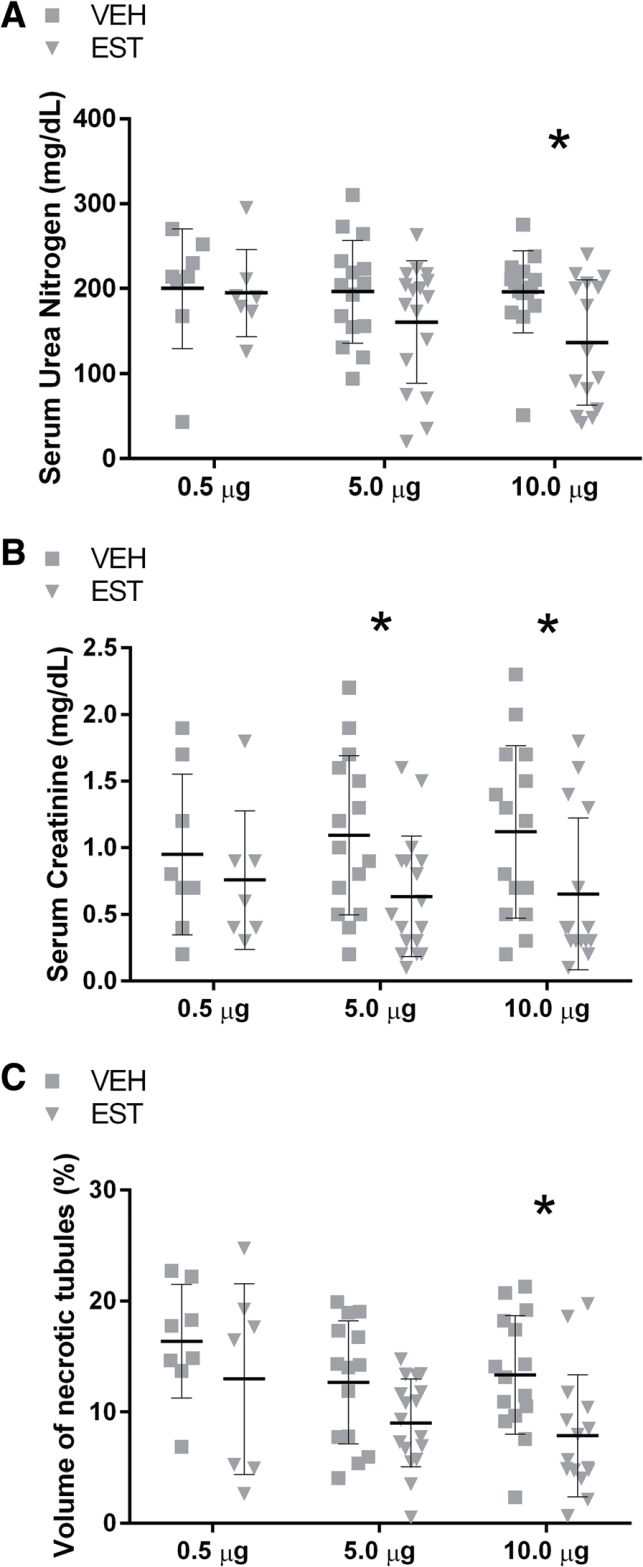
17β-estradiol exerted a dose-dependent renoprotective effect after cardiac arrest and cardiopulmonary resuscitation (CA/CPR) in young males. Blood and tissue samples were collected 24 h after CA/CPR in young male intravenously injected with 0.5, 5, or 10 μg of 17β-estradiol or vehicle 15 min after return of spontaneous circulation. Post-arrest administration of 17β-estradiol exerted a dose-dependent renoprotective effect. At 0.5 μg, 17β-estradiol administration exerted no apparent effect on renal injury 24 h after CA/CPR. Five micrograms caused significant reduction in serum creatinine (**b**) but not serum urea nitrogen (**a**) and necrotic tubules (**c**). At a dose of 10 μg, 17β-estradiol significantly reduced serum urea nitrogen (**a**), serum creatinine (**b**), and necrotic tubules (**c**) by 41 %, 30 %, and 31 %, respectively. Data are presented as mean ± standard deviation, n = 8–15, *P < 0.05 by two-way analysis of variance with *post hoc* Sidak’s test. *EST* 17β-estradiol-treated mice, *VEH* vehicle-treated mice

To determine the effect of acutely injected exogenous estrogen on AKI, male young mice were subjected to 8 min of CA and then resuscitated. Fifteen minutes after ROSC, 0.5, 5, or 10 μg of 17β-estradiol or vehicle was injected intravenously. Figure 2 summarizes the findings of the dose-escalation study in young male mice. Estrogen dose-dependently reduces renal injury. Ten micrograms of 17β-estradiol reduced sCr, sUN, and VNT by 30 %, 41 %, and 31 %, respectively. Therefore, this dose was selected for the continuation of the study.

### Renal blood flow is not altered by estrogen administration following CA/CPR

**Fig. 3:**
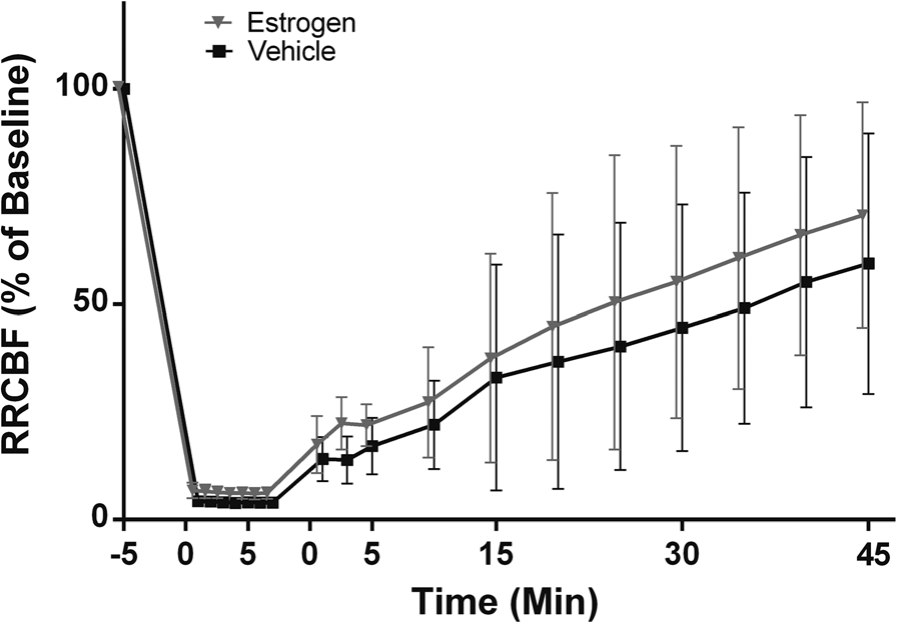
Regional renal cortical blood flow (RRCBF) is not altered by estrogen administration. Laser Doppler flow of RRCBF was measured in the peri-arrest period for each animal treated with 17β-estradiol or vehicle at 15 min after return of spontaneous circulation (ROSC). The average of the measurement taken 5 min before and immediately before cardiac arrest (CA) served as baseline flow, and all measurements were described as percentage of baseline flow to minimize variation between animals. RRCBF values are not affected by administration of 17β-estradiol. RRCBF dropped immediately to nonperfusing values on CA. RRCBF remained low after ROSC for 15 min and then gradually returned to the baseline in the 45-min follow-up period. There was no significant difference in RRCBF between estrogen-treated and vehicle-treated animals at any point. Data are presented as mean ± standard deviation, n = 6 or 7 per group

To evaluate potential acute systemic effects of estrogen, we measured RRCBF during and after CA/CPR and collected blood samples for arterial blood gas analysis in separate cohorts. Figure 3 and Table 1 summarize the results of experiments to measure RRCBF and metabolic parameters in the immediate period following CA/CPR and estrogen administration. There were no significant differences in RRCBF or metabolic parameters between groups.

**Table 1:** Post-CA/CPR arterial metabolic values

Treatment	pH	PCO_2_	pO_2_	HCO_3_^−^	Hb	K^+^	Na^+^	Lac	Ca^++^	BE
Estrogen	6.9 (0.1)	47.0 (16.9)	226 (127)	9.8 (3.2)	13.6 (0.5)	5.1 (0.6)	145 (2.6)	8.7 (2.9)	1.1 (0.1)	−19.8 (4.3)
Vehicle	6.8 (0.2)	54.1 (19.4)	141 (104)	8.6 (3.7)	14.4 (1.0)	5.8 (0.7)	147 (3.8)	10.3 (4.5)	1.1 (0.1)	−22.4 (5.3)

Data are presented as mean (standard deviation)

*CA/CPR* cardiac arrest and cardiopulmonary resuscitation

### Sex difference in the severity of AKI after CA/CPR and the effect of post-arrest administration of 17β-estradiol

**Fig. 4:**
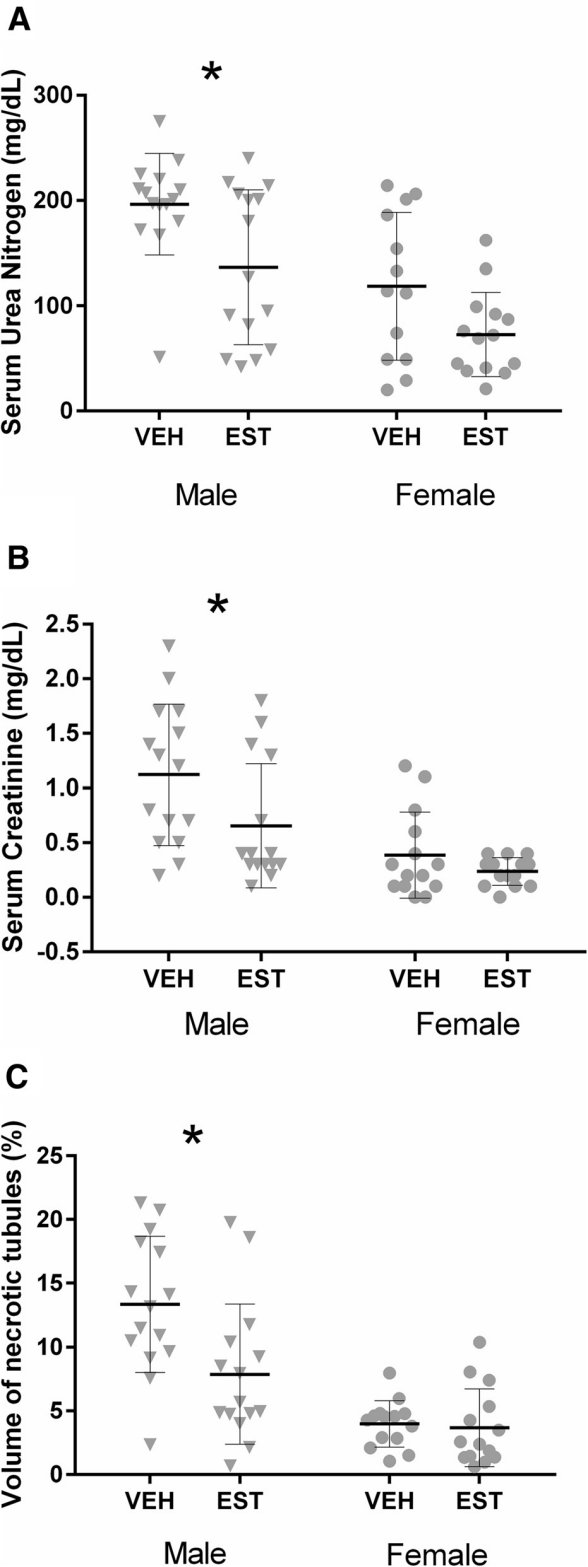
Sex difference in the severity of acute kidney injury after cardiac arrest and cardiopulmonary resuscitation (CA/CPR) and the effect of post-arrest administration of 17β-estradiol. Blood and tissue samples were collected 24 h after CA/CPR in young (10–15 weeks) male or female mice intravenously injected with 10 μg of 17β-estradiol or vehicle 15 min after return of spontaneous circulation. There was a sex difference in estrogen-mediated amelioration of renal injury following CA/CPR in young mice. Estradiol treatment significantly reduced serum urea nitrogen (**a**), serum creatinine (**b**), and tubular cell death (**c**) in males (*triangle*), but young females were not affected, because injury was small (*circle*). Data are presented as mean ± standard deviation, n = 14 or 15 per group, *P < 0.05 by two-way analysis of variance with *post hoc* Sidak’s test. *EST* 17β-estradiol-treated mice, *VEH* vehicle-treated mice

**Fig. 5:**
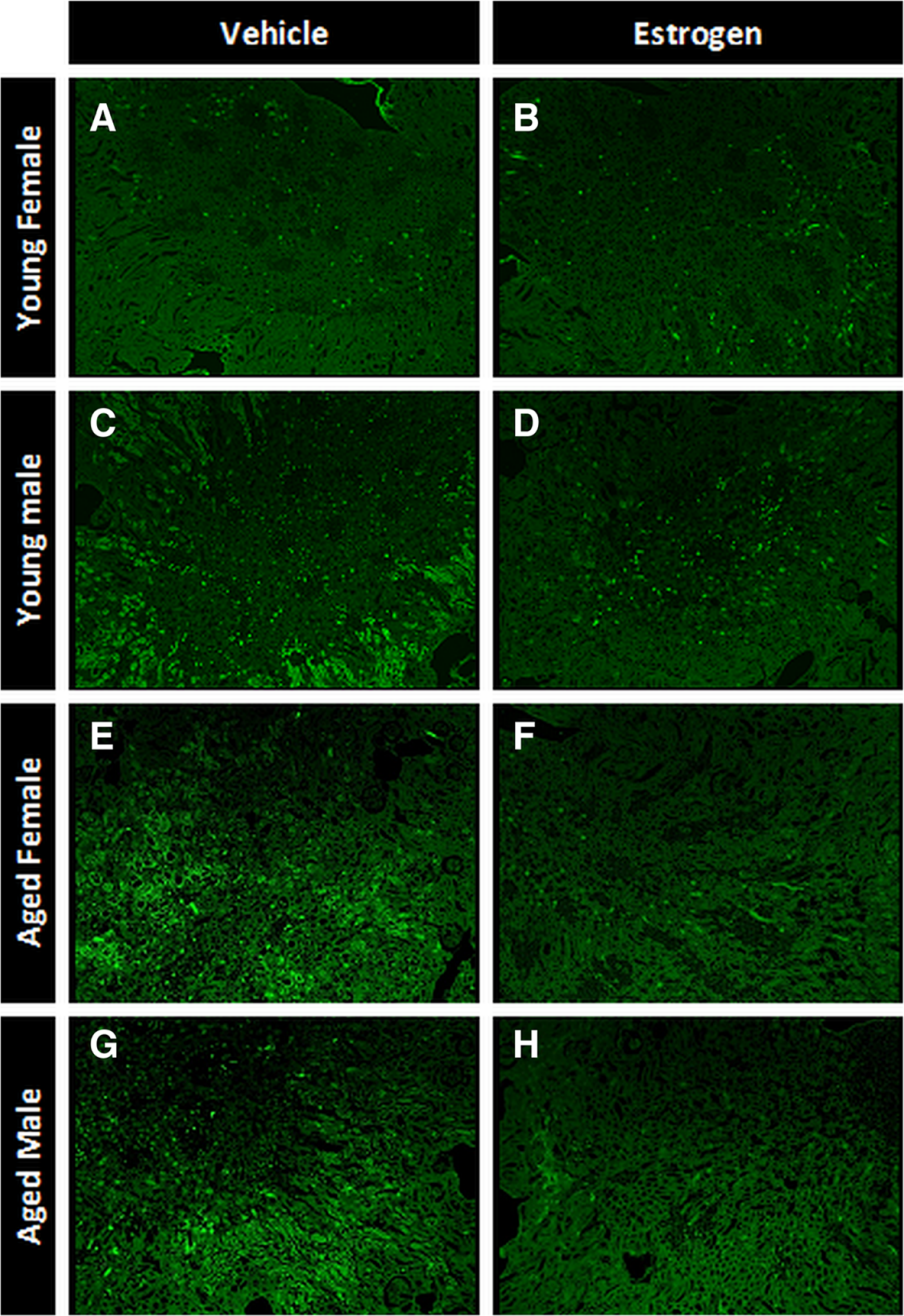
Representative photomicrographs of Flouro-Jade B-stained kidney section 24 h after cardiac arrest and cardiopulmonary resuscitation. Mouse kidneys were perfusion-fixed and removed 24 h after cardiac arrest and cardiopulmonary resuscitation and then stained with Flouro-Jade B, which stains early necrotic cells bright green. **a** Young female vehicle. **b** Young female estrogen. **c** Young male vehicle. **d** Young male estrogen. **e** Aged female vehicle. **f** Aged female estrogen. **g** Aged male vehicle. **h** Aged male estrogen

To investigate whether there are sex differences in the effect of acutely administered estrogen on AKI after CA/CPR, young male and female mice were subjected to CA/CPR and administered 10 μg of either 17β-estradiol or vehicle 15 min after ROSC. Table 2 summarizes the baseline and resuscitation data for this protocol. Females required slightly greater resuscitation time and epinephrine than males, but the mortality was not different (time to resuscitate: 123 ± 33 versus 155 ± 30 sec, *P* < 0.05; epinephrine dose: 0.52 ± 0.1 versus 0.73 ± 0.1 μg/g body weight, *P* < 0.05; mortality: 32 % versus 26 %, *P* = 0.58, n = 30 and 28, males versus females). Figure 4 illustrates the effect of estrogen on AKI in both sexes, and Fig. 5 presents representative histologic images. Males suffered greater injury than females, and sex represented 23 % of the total variation in sUN, 25 % in sCr, and 35 % in VNT. Estrogen reduced injury, representing 13 % of the total variation in sUN, 7.2 % in sCr, and 6.4 % in VNT. *Post hoc* comparison demonstrates that estrogen is renoprotective in males only. Thus, in young male mice, acutely injected estrogen after ischemic insult ameliorates AKI. Young female did not suffer renal injury after 8 min of CA and were not affected by the post-arrest administration of estrogen.

**Table 2:** Baseline and resuscitation data according to age, sex, and treatment

Group	Treatment	Number^a^	Age, week	Body weight, g	Epinephrine dose, μg/g body weight	Epinephrine dose, g	CPR time, s	Survival rate, %
Young male	VEH	15	11.1 (0.4)	25.0 (1.6)	0.53 (0.09)	13.2 (2.0)	129 (29)	68
	EST	15	11.2 (0.4)	25.1 (1.8)	0.51 (0.11)	12.7 (2.2)	118 (37)	68
	*P* ^*^		0.90	0.97	0.09	0.71	0.42	1
Young female	VEH	14	12.5 (2.0)	19.7 (0.8)	0.75 (0.08)	14.8 (1.6)	158 (28)	64
	EST	14	12.9 (1.7)	19.9 (1.1)	0.71 (0.11)	14.0 (1.9)	151 (33)	88
	*P* ^*^		0.58	0.94	0.99	0.42	0.75	0.10
Aged male	VEH	8	83.8 (2.9)	34.9 (6.8)	0.42 (0.10)	14.3 (1.9)	136 (36)	71
	EST	10	82.6 (3.1)	39.2 (8.6)	0.36 (0.09)	13.6 (1.5)	138 (27)	67
	*P* ^*^		0.26	0.26	0.11	0.33	0.87	0.60
Aged female	VEH	9	81.4 (2.4)	29.1 (5.1)	0.51 (0.13)	14.2 (1.9)	142 (35)	100
	EST	10	82.2 (1.4)	31.1 (4.3)	0.42 (0.09)	12.9 (2.1)	130 (35)	83
	*P* ^*^		0.38	0.33	0.11	0.16	0.44	0.49

Data are presented as mean (standard deviation)

*CPR* Cardiopulmonary resuscitation, *VEH* vehicle, *EST* 17β-estradiol

^a^number of survives

*P values shown for Sidak’s multiple comparison test or chi-squared test when appropriate

### Estrogen is acutely renoprotective in old mice of both sexes

**Fig. 6:**
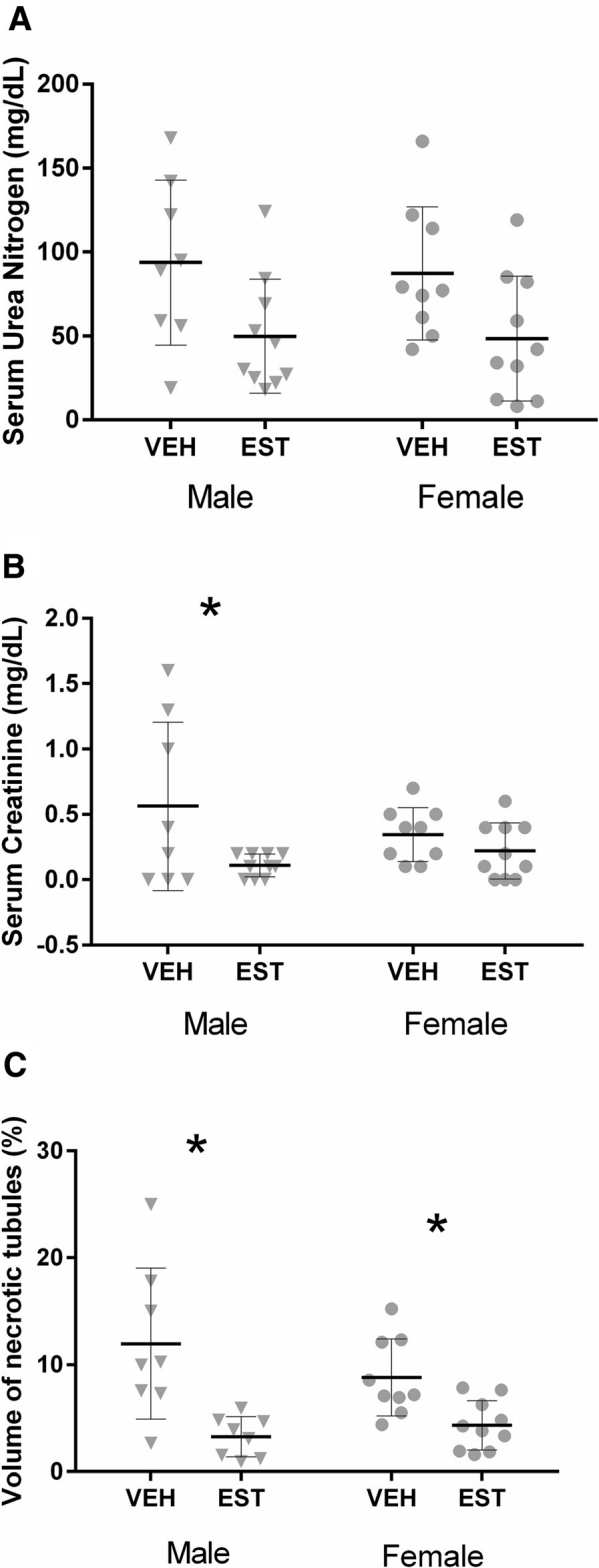
17β-estradiol after cardiac arrest and cardiopulmonary resuscitation (CA/CPR) ameliorates renal injury in old mice of both sexes. Blood and tissue samples were collected 24 h after CA/CPR in aged (78–87 weeks) male or female mice intravenously injected with 10 μg of 17β-estradiol or vehicle 15 min after return of spontaneous circulation. Unlike in young mice, in this group of mice at advanced age, there was no significant effect of sex on renal injury. Estradiol treatment significantly reduced serum creatinine (**b**) and tubular cell death (**c**) in aged males (*triangle*) and tubular cell death (**c**) in aged females (*circle*). Serum urea nitrogen was not affected by estrogen treatment in either aged males or aged females (**a**). Data are presented as mean ± standard deviation, n = 8–10 per group, *P < 0.05 by two-way analysis of variance with *post hoc* Sidak’s test. *EST* 17β-estradiol-treated mice, *VEH* vehicle-treated mice

To address the effect of aging on the renoprotective effect of estrogen according to sex, we tested 17β-estradiol administration in aged mice in both sexes. Eight-minute CA/CPR was 100 % lethal in mice of extreme age (n = 7). Therefore, we reduced the CA time from 8 to 7 min. Seven-minute CA/CPR in aged mice was associated with similar resuscitation and mortality to 8 min CA/CPR in young mice (age: 83 ± 2.6 versus 12 ± 1.5 weeks; time to resuscitate: 136 ± 32 versus 138 ± 35 sec, *P* = 0.78; mortality: 21 % versus 29 %, *P* = 0.32, n = 47 and 82, aged versus young). There was no sex difference in these parameters (time to resuscitate: 137 ± 31 versus 135 ± 34 sec, *P* = 0.86; mortality: 30 % versus 10 %, *P* = 0.07, n = 26 and 21, males versus females). These resuscitation data are detailed in Table 2. Figure 6 illustrates the effect of estrogen on AKI in both sexes in aged mice. Overall, in aged mice, estrogen significantly reduced sUN, sCr, and VNT (representing 23 %, 16 %, and 40 % of total variation, respectively). *Post hoc* comparison showed significant reduction of sCr (80 %) and VNT (73 %) in aged males. In this protocol, females treated with 17β-estradiol demonstrated reduced VNT relative to vehicle (a 51 % reduction, *P* < 0.05), and a protective trend was evident in sUN (45 %, *P* = 0.08). Therefore, in mice of advanced age, there was no significant effect of sex on renal injury.

### Serum estrogen levels in aged female mice are the same as physiologic levels in male mice 24 h after CA/CPR

We measured serum estradiol levels in estrogen- and vehicle-treated animals 24 h after CA/CPR to determine whether prolonged differences in estradiol serum levels could contribute to renal outcome. In young female mice, serum levels of estradiol varied considerably in a manner consistent with reproductive cycling (62 ± 123 pg/ml, n = 23). Serum estradiol concentration was negligible in aged females (16 ± 9 pg/ml, n = 17, *P* < 0.05 versus young females) and the same as that in young and old males (17 ± 10 and 11 ± 8 pg/ml, n = 30 and 17). Serum estradiol was not different 24 h after treatment whether the animals were treated with 17β-estradiol or vehicle, suggesting that it returned to physiologic levels prior to 24 h after administration.

### Estrogen receptor expression is upregulated in aged females compared with young females 24 h after CA/CPR

**Fig. 7:**
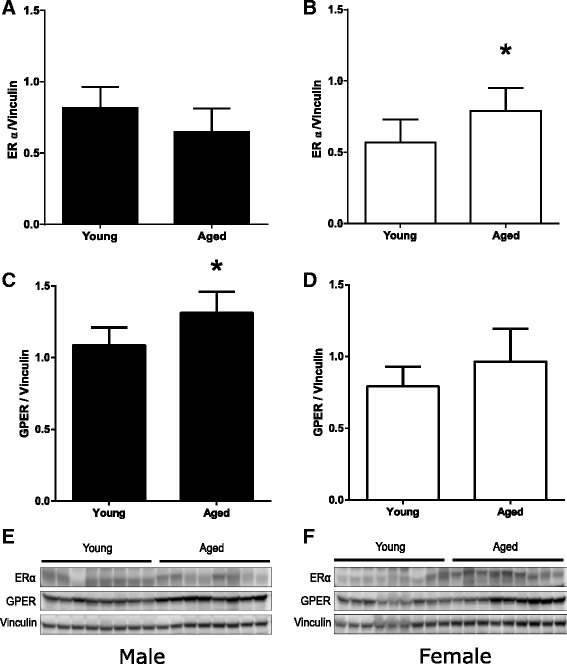
Renal expression of ERα and GPER 24 h after cardiac arrest and cardiopulmonary resuscitation (CA/CPR). Aging is associated with increased renal expression of ERα in female mice and GPER in male mice 24 h after CA/CPR. ERα and GPER expressions were determined in kidney homogenates of young (10–15 weeks) or aged (78–87 weeks) male (**a**, **c**, **e**) or female (**b**, **d**, **f**) mice harvested after 24 h of CA/CPR by Western blotting with subsequent quantification by densitometry. Densitometric analyses for ERα and GPER are shown in (**a**–**d**). Western blots including homogenates from all animals (n = 8 or 9 per group) are shown in (**e**, **f**). Note the increase in ERα abundance in aged females (**b**, **f**) and increase in GPER abundance in aged males (**c**, **e**). Data are presented as mean ± standard deviation, n = 8 or 9 per group, *P < 0.05 by unpaired *t* test. *ER* estrogen receptor, *GPER* G protein-coupled estrogen receptor

To elucidate the mechanism of the observed difference in effect of post-arrest estrogen between young and old females and between males and females, we measured levels of the two ERs expressed in renal tissue [27, 28] (ERα and GPER) after CA/CPR. In aged females, the expression of ERα was increased compared with young females (*P* = 0.01, Fig. 7b, f). By contrast, aged males demonstrated a significant increase in the expression of GPER compared with young males (*P* < 0.01, Fig. 7c, e).

## Discussion

There are three major findings of the present study. First, the advantage of female sex against AKI after whole-body ischemia, which is consistently observed both in our previous study and in the present study, weakens with age. Second, estrogen exerts renoprotective effects even when administered after resuscitation from CA. To the best of our knowledge, this is the first study to demonstrate that exogenous estrogen acts as a potent renoprotective agent when administered in a clinically relevant manner, after whole-body ischemia-reperfusion. Third, estrogen injection improves renal outcome in males, but reproductively intact young female are not affected because injury is small, whereas in aged mice, estrogen is beneficial to both sexes. Taken together, these three findings indicate that post-arrest estrogen administration is renoprotective in a sex- and age-dependent manner.

We confirmed that females enjoy relative renoprotection after CA as previously reported [12, 13, 21] and extend this finding with the finding that this advantage weakens in middle age as tubular necrosis becomes equivalent to that of males. Functional measures of injury are still reduced in middle-aged females. Previous studies have demonstrated that female mice enter reproductive senescence by 44–64 weeks [29]. We chose to assess sex difference in middle age precisely because some females, but not all, may have ceased reproductive cycling, thus the mixed finding was expected. To further evaluate the effect of age and reproductive cycling, we used mice of extreme age (78–87 weeks). These aged females lose their advantage both in renal function and in tubular cell death. Serum estradiol levels in aged females decreased to the same level as physiologic level of male mice. Taken together, these data suggest that females lose renoprotection according to age because of changing exposure to estrogen. This would recapitulate our previous findings using ovariectomized young mice [12, 13]. However, whether intact females lose their advantage according to reproductive senescence remains a vital translational question, and we directed this study at this query. Our findings are in line with previous literature studying age-dependent progression of renal damage in female mice. Urbieta-Caceres et al. suggested that aging-induced renal interstitial fibrosis in female mice was mediated by loss of endogenous estrogen [30]. Zheng et al. reported that female mice developed progressive glomerulosclerosis after menopause [31]. Although we did not find any difference between young and aged female surgically naïve animals, those structural abnormalities caused by aging may increase susceptibility to acute ischemic insult in aged females.

The present study demonstrates for the first time a beneficial effect of estrogen on AKI after CA/CPR in male mice. To assess critical translational benefit of estrogen in male mice, we chose the timing of injection at 15 min after ROSC and evaluated three doses. We chose CA/CPR as our model for ischemia-reperfusion injury because it replicates key features of clinical ischemia-reperfusion, making it clinically relevant. First, it replicates the most common cause of clinical AKI, whole-body hypoperfusion [4]. Second, as can be seen from our data, CA/CPR produces mild-moderate renal injury similar to that seen in clinical ischemia-reperfusion injury. Third, other groups have found that CA/CPR may more closely model ischemic inflammatory, immune, and renal arterial flow changes than occlusion of the renal pedicle, a widely used model of renal ischemia [32, 33]. Our data indicate a dose-dependent reduction of renal injury by post-ischemic administration of estrogen. In previous experiments using young female mice exposed to CA/CPR, we found that ovariectomy significantly increased injury and that estradiol treatment 7 days prior to ischemic insult, designed to replicate physiologic levels, restored protection [12, 33]. Müller et al. found that increased mortality after focal renal ischemia in male rats compared to female was reversed with estrogen treatment 7 days prior to the insult [10]. Park et al. also reported that estrogen treatment 15 days before renal artery occlusion reduces the post-ischemic increase of sUN and sCr in male mice [11]. Takaoka et al. [34] and Tanaka et al. [35] demonstrated with the renal pedicle occlusion model that male rats injected with estrogen 15 min prior to reperfusion demonstrated improved post-ischemic renal outcomes. On the other hand, a recent study which employed intramuscular injection of estrogen 3 days prior to renal ischemia-reperfusion surgery found that estrogen’s protective effect was limited to ovariectomized female rats and did not protect male rats [36]. The discrepancy of these findings is probably due to the heterogeneity of the experimental model and the length of time of administration. Estrogen dose varied according to the study (25 μg/kg × 7 days [10], 40–500 μg/kg × 14 days [11], 100 μg/kg × single dose [35] and 500 μg × 3 days [36]); however, no prior study has evaluated the effect of estrogen administration *after* reperfusion, either whole-body ischemia/reperfusion or renal vascular occlusion. In the present study, only higher dose of estrogen (400 μg/kg) injected during the early reperfusion phase improved the renal outcome in male mice. Estrogens may exert a different mechanism of action depending on the timing of injection, dose, and extent of overall injury. Thus, it is notable that not all studies of estrogen in CA have demonstrated a protective effect. A recent study using a male rat model of ventricular fibrillation and resuscitation reported that estrogen administration did not result in beneficial myocardial effect or impact survival. The survival rates 3 h after resuscitation in this severe-injury model are 0 % versus 33 %, *P* = 0.32, estrogen versus saline, for bolus injection and 63 % versus 100 %, *P* < 0.05, estrogen versus saline, for continuous injection [37]. The present study uses a CA/CPR model which demonstrates a mild injury and survival of more than 65 % in order to assess renal outcome 24 h after ROSC, and this may explain the apparently discordant result.

Pre-ischemic estrogen alters outcome of focal renal ischemia via nitric oxide and endothelin-1 [10, 34]. Because both of these mediators are believed to act on renal blood flow, our findings that neither RRCBF nor systemic metabolic parameters are affected by estrogen during the post-arrest period suggest that the mechanism of acute renoprotection by post-arrest estrogen may be distinct from that of chronic or pre-ischemic administration. Since no prior study addresses post-treatment with estrogen in the kidney, we note that after experimental stroke estrogen exerted a protective effect due to enhanced cerebral blood flow [38, 39]. In our whole-body ischemia model, we found that estrogen does not alter RRCBF or systemic metabolic parameters in the recovery period up to 45 min following CA/CPR. We cannot eliminate the possibility that renal medullary but not renal cortical blood flow is affected by estrogen, as it is reported that even though cortical blood flow fully recovers in 30–45 min after bilateral occlusion of the renal arteries and veins and reperfusion, medullary blood flow remains compromised for prolonged periods that are associated with a long-term decline in renal function [40, 41]. However, this investigation is focused on acute, 24-h outcomes, and estrogen mediation of long-term outcome was not assessed in this study. Another possibility is that the use of isoflurane in our study confounds the evaluation of blood flow, as it is a vasodilator. An additional possible limitation regarding the use of isoflurane is its well-characterized renoprotective effect [42]. It is not possible to perform our study without anesthesia; we carefully designed experiments to exclude differential effect of either vasodilatory or renoprotective properties of isoflurane. As all animals received the same concentration of isoflurane, no observed effect can be due to the influence of isoflurane alone.

In the present study, efficacy of post-ischemic estrogen injection was observed amongst young mice only in males. On the other hand, estrogen was renoprotective in old mice of both sexes. In our previous studies, we found that deletion of ERs α, β, and GPER did not interfere with protection against renal injury from ischemia/reperfusion when estrogen was administered for a week prior to CA/CPR [12, 13]. In evaluating these findings, we hypothesized that pre-ischemic and repeated dosing of estrogen could protect the kidney through multiple mechanisms mediated by more than one receptor; thus, deletion of a single receptor would not abrogate the protective effect. As acute and chronic administrations of estrogen are differentially protective in neurologic ischemia [43], this might also be the case in renal ischemic injury. Thus, it is essential to evaluate involvement of renal ERs in the effect we observed. Since aged females are in a low-estrogen environment compared with young females, we hypothesized that ER expression levels differ between the two, and this might explain the different response to the exogenous estrogen. We therefore measured renal ERα and GPER levels in animals not treated with estrogen. We found that the expression of ERα in aged females was increased compared with young females 24 h after CA/CPR. This finding in animals after CA/CPR is similar to those of others: in surgically naïve animals, ERα expression increases with age in renal tissue [44]. Our finding is limited in that the 24-h time point may not precisely replicate the levels present at the time of reperfusion; therefore, the inference of mechanistic association should be interpreted with caution. In heart [45] and brain [46, 47], ER activation reduces ischemic injury. In light of these findings and in light of recent data indicating ERα as a mediator of protective estrogen signaling in female rats with pulmonary hypertension-induced right ventricular failure [48], we speculate (with the above limitations) that protective acute estrogen effects in aged females are mediated at least in part by ERα, and further investigation will test this hypothesis.

Sex differences in the expression of ER during aging [44] and in neuroprotective effects of ER agonists have been reported [49]. Broughton et al. found that GPER expression was increased in the brain of male mice but not of female mice after transient focal cerebral ischemia [50] and that GPER activation worsens outcome in males but that an opposite protective effect occurs in females [49]. Interestingly, the expression of GPER in kidney 24 h after CA/CPR was increased in aged male compared with young male mice in our study. The underlying mechanisms responsible for the estrogen’s protective effect to renal ischemic injury observed in our study require further investigation, particularly with regard to sex difference. Androgens, including testosterone, are reported to increase the susceptibility of males to ischemic renal injury [10, 11]. Myocardial ischemia reperfusion injury in male rats aggravated cardiac damage through androgen receptor [51]. The interaction of sex hormones besides estrogen and their change in the aging process might modify the effect of estrogen’s renoprotection in males. Together, these findings highlight the complex nature of endogenous estrogen signaling, which may vary between different physiological and disease settings. Sex should be considered in identification of the underlying mechanism responsible for the effect of estrogen.

In addition to the sex and age specificity of estrogen’s renoprotective mechanisms, it is possible that estrogen exerts its effect differently because of timing. We acknowledge that most previous studies are designed in the aspect of hormone replacement therapy, mostly involving long-term drug pretreatment before experimental ischemia, and that more work is required to fully evaluate the efficacy of estrogen as a potent therapeutic agent. Our study, designed to evaluate efficacy of acute and transient estrogen exposure, shows that the renoprotective effect is rapid, exerted within 24 h of ischemia reperfusion, perhaps sooner, suggesting one of the non-nuclear, rapid actions of estrogen [45, 52]. The fact that serum estradiol levels have returned to physiologic levels within 24 h after estradiol injection reinforces the argument of estrogen’s rapid action. In our recent *in vitro* study, we found that hyperpermeability of glomerular endothelial cells after ischemia was prevented by estrogen in part through GPER [21]. Interestingly, in that study, the significant decrease in glomerular permeability by estrogen observed after 4 h of re-oxygenation was no longer evident at 8 h, suggesting that estrogen’s effect is acute and transient. Preserved glomerular barrier integrity during the acute phase of reperfusion might prevent subsequent tubular dysfunction and tubular cell death. We did not assess whether post-arrest estrogen injection alters glomerular permeability or GPER expression at an early phase of reperfusion in the present study, which was focused on whether estrogen exerted any acute beneficial effect in renal ischemia. Further investigation of glomerular permeability effects of estrogen *in vivo* might delineate a mechanism of the protective effect we observed. We did not find any difference in 24-h survival rate between estrogen-treated animals and vehicle-treated animals in our potassium-induced CA model (76 % versus 66 %, *P* = 0.30, estrogen versus vehicle), and estrogen-treated animals had less kidney injury.

## Conclusions

In summary, we report that estrogen injection shortly after resuscitation from CA ameliorates renal injury in young males. Young females were not protected by estrogen, because of reduced renal injury from CA/CPR itself relative to males and aged females. In aged females, the protective effect of exogenous estrogen may be recovered with loss of endogenous estrogen.

The magnitude of the effect in our model offers promise for future translational study. For successful translation, an observed preclinical effect should match with the population at risk for the disease. Our study suggests that acute, estrogen-mediated renoprotection is selective to males and aged females, precisely the population at risk for clinical AKI. Our findings suggest that further investigation of estrogen-mediated renoprotection should focus on rapid effects and whether in aged animals these are mediated thorough cognate ERs.

## Key messages

- Aging reduces the female advantage in renal outcome after whole-body ischemia reperfusion injury.
- When given in a clinically relevant manner *after* cardiac arrest and cardiopulmonary resuscitation, estrogen reduces renal injury in male and aged female mice.
- This study provides early evidence that efforts to translate this first demonstration of an estrogenic acute post-resuscitation renoprotective effect may best be focused on rapid effects which in the aged may be mediated by cognate estrogen receptors.

## Abbreviations

AKI: Acute kidney injury
BSA: Bovine serum albumin
CA: Cardiac arrest
CPR: Cardiopulmonary resuscitation
EKG: Electrocardiogram
ER: Estrogen receptor
GPER: G protein-coupled estrogen receptor
HRP: Horseradish peroxidase
ROSC: Return of spontaneous circulation
RRCBF: Regional renal cortical blood flow
sCr: Serum creatinine
sUN: Serum urea nitrogen
TBST: Tris-buffered saline with Tween-20
VNT: Volume of necrotic tubules
